# Beyond Time Saved: A Framework for Comprehensive Evaluation of AI Clinical Documentation

**DOI:** 10.2196/86091

**Published:** 2026-10-27

**Authors:** Ekstrom Victoria Sze Min

**Affiliations:** 1 Department of Gastroenterology and Hepatology, Singapore General Hospital, 20 College Rd, Singapore, 169856, Singapore, 65 63214684; 2 Narratives in Medicine, SingHealth Duke NUS Medical Humanities Insititute, Singapore, Singapore

**Keywords:** clinical documentation, ambient scribes, patient-clinician communication, narrative medicine, health equity, implementation science, automation bias, clinical reasoning

## Abstract

Ambient AI scribes have been rapidly adopted across health care organizations to mitigate documentation burden and burnout. Initial single-system reports described substantial time savings, but more recent multisite evaluations and meta-analyses describe modest, heterogeneous effects with generally low methodological quality. Most evaluations emphasize efficiency and satisfaction, leaving potential effects on communication quality, narrative preservation, cognitive performance, and equity largely unmeasured. This viewpoint aimed to characterize evaluation patterns in prominent ambient AI scribe implementations, explain why unmeasured domains warrant investigation for patient safety and care quality, and propose an expanded framework with feasible research priorities. This analytic viewpoint mapped evaluation domain coverage in a purposive, illustrative sample of prominent implementation reports published through April 2026, then synthesized findings with literature on clinical communication, narrative medicine, automation effects, and health equity. Reporting was guided by Scale for the Assessment of Narrative Review Articles (SANRA) principles for narrative reviews; PRISMA (Preferred Reporting Items for Systematic Reviews and Meta-Analyses) was not used because the work is a domain-coverage characterization rather than an evidence synthesis estimating effects. A single reviewer (VSME) performed selection and extraction, consistent with the viewpoint’s illustrative intent. Across the included implementations, evaluation consistently measured documentation time, after-hours work, and clinician satisfaction. Within this illustrative sample of 6 independently peer-reviewed real-world implementation evaluations, we did not identify any that reported systematic assessment of patient-clinician communication using validated instruments, structured comparison of AI-generated against human-generated notes for narrative fidelity, longitudinal evaluation of cognitive or educational effects, or equity analyses stratified by language and demographic group. The absence in this purposive sample reflects an absence of public reporting rather than proof that no organization has measured these dimensions internally. Limited error analyses found frequent omissions, with accuracy declining as encounter complexity increased. Evidence from multilingual and interpreter-mediated encounters was notably sparse. Current evidence supports continued implementation with quality safeguards in settings where benefits are demonstrated locally rather than universal adoption on the strength of early single-system reports. Organizations implementing ambient AI scribes should consider embedding prospective evaluation across 4 domains: validated communication quality measurement, structured narrative content comparison, longitudinal cognitive and training studies, and equity analyses across languages and demographics. Three near-term priorities offer immediate feasibility for organizations with evaluation capacity: (1) deploying validated communication instruments such as the Communication Assessment Tool or Consumer Assessment of Health Care Providers and Systems (CAHPS) within ongoing rollouts; (2) launching multisite longitudinal trainee cohorts comparing programs with and without ambient AI exposure; and (3) oversampling multilingual and interpreter-mediated encounters for stratified equity analyses. Narrative preservation requires foundational methodological work before implementation-ready measures exist. Editors, implementers, and vendors can each act now: editors by requiring domain-coverage reporting; implementers by preregistering evaluation plans before rollout; vendors by publishing stratified performance data as a routine condition of deployment. The goal is to preserve documented efficiency gains while generating evidence about communication, narrative, reasoning, and equity dimensions that current evaluations leave unmeasured.

## Introduction

Clinical documentation burden represents a significant workforce crisis in modern medicine. Nearly half of US physicians report burnout symptoms, contributing an estimated US $4.6 billion annually in costs from turnover and reduced clinical hours [1]. Physicians spend approximately 2 hours on electronic health record tasks for every hour of direct patient care [2], often extending 1 to 2 hours into evenings [3]. This burden links directly to patient safety, as burnout correlates with higher self-reported medical errors [4] and poor electronic health record usability independently associates with increased burnout [5].

Ambient AI scribes have been rapidly adopted to address this crisis [6]. Initial reports described substantial efficiency and well-being gains, with reductions in documentation time and burnout rates [7]. More recent evidence is more measured. A multisite study found only modest decreases in total electronic health record time and documentation time alongside a modest increase in weekly visit volume [8], and a systematic review and meta-analysis of 23 studies [9] demonstrated a moderate pooled reduction in documentation workload across 14 studies (standardized mean difference −0.71; 95% CI −0.93 to −0.49) but considerable heterogeneity across studies and generally low methodological quality. The effect of ambient AI scribes on documentation workload appears real but variable, setting-dependent, and supported by an evidence base in which study quality remains an important limitation. Current evaluation frameworks focus predominantly on these efficiency metrics, creating evidence gaps concerning domains central to patient safety and care quality.

Systematic error analyses remain rare. One instrument-validation study comparing AI-generated drafts against reference standards found errors that must be evaluated to mitigate safety risks [10]. Accuracy degrades as clinical complexity increases [11]. Evidence is largely absent for performance in multilingual or interpreter-mediated encounters. Ambient AI scribes combine automatic speech recognition, speaker diarization, and large-language-model summarization [12,13], and inequities may arise at any of these stages. Underlying automatic speech recognition components alone show significant accuracy disparities across racial and dialect groups [14]. These are component-level findings, not direct evidence of ambient-scribe inequity; the downstream note reflects compounded effects from across the pipeline. A staged audit that follows the pipeline—word error rates by language, accent, and dialect group first; speaker attribution accuracy second; clinical-concept and narrative-relevant omission third—is feasible with existing tools and is the analytic foundation for equity claims about full ambient-scribe systems. Documentation automation bias is the analogous concern at the synthesis stage: clinicians may accept an AI-generated narrative that then shapes downstream reasoning, coding, orders, and the medico-legal record. Plausible failure modes include a missed red-flag symptom in the history of present illness, an inflated problem list, or an incorrect speaker attribution that quietly redirects the differential. The mechanism is well characterized in the health care automation bias literature for decision support [15]; how it translates when the AI output is the encounter narrative rather than a discrete recommendation is an open empirical question. Direct measures of patient experience remain largely absent from this literature [16]. Errors and omissions in clinical documentation propagate through downstream care: notes guide handoffs, coding, continuity decisions, and the medicolegal record on which subsequent clinicians depend [17].

Given documented time savings and reduced burnout, ambient AI scribes rank among the most swiftly adopted clinical AI technologies [7,18]. At this scale, failing to systematically assess impacts on care quality leaves a set of plausible, testable risks unexamined: that patient-clinician communication may be subtly altered, that clinical narratives important for care coordination may be degraded, and that documentation accuracy may differ across patient populations [19,20]. We do not claim these harms are established for ambient scribes; we argue they are plausible and measurable using existing instruments, on the strength of adjacent evidence on clinical communication, automation effects, electronic health record narrative quality, and speech-recognition equity.

This viewpoint characterizes current evaluation patterns in prominent ambient AI scribe implementations, explains why unmeasured domains warrant investigation for care quality, and proposes an expanded assessment framework guiding future research and responsible adoption. To organize the analysis, we present a conceptual model (Figure 1) describing how ambient AI scribes may affect care quality through 2 parallel pathways: the presence of a recording device in the clinical encounter, which modifies in-encounter behavior, and automated draft generation, which reshapes the resulting note and the clinician’s review task. Each pathway has plausible benefits and risks across the 4 evaluation domains we consider, communication, narrative, reasoning, and equity, with example measurable indicators that anchor the proposals that follow.

**Figure 1.**
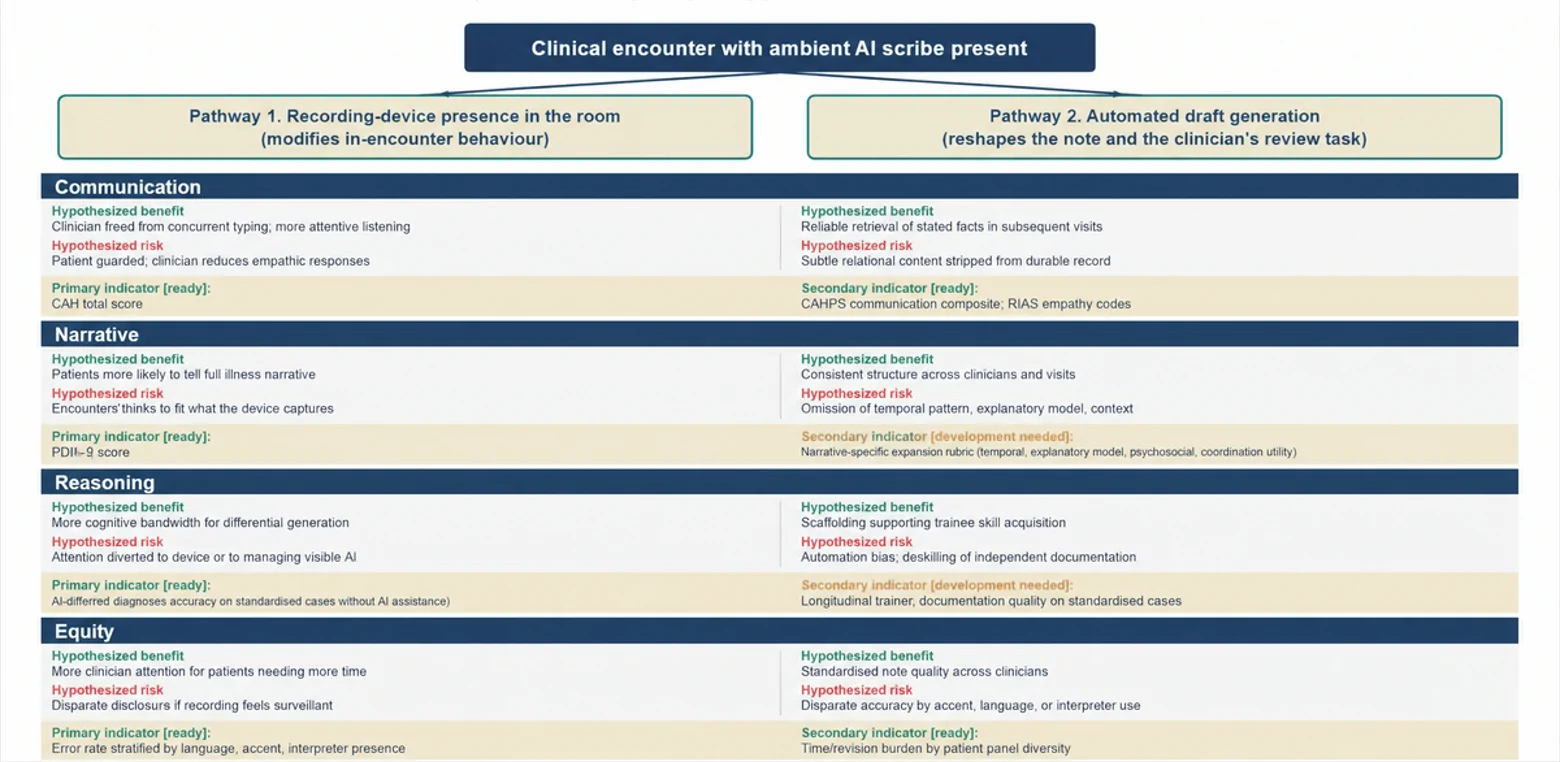
Conceptual model: pathways by which ambient AI scribes may affect care quality, mapped to 4 evaluation domains. CAH: Consumer Assessment of Health care; CAHPS: Consumer Assessment of Health care Providers and Systems; PDQI-9: Physician Documentation Quality Instrument; RIAS: Roter Interaction Analysis System.

## Scope and Analytic Approach

We conducted a purposive, analytic review characterizing evaluation patterns in ambient AI scribe implementations rather than a systematic review. The aim was to map evaluation domain coverage in a purposive, illustrative sample of independently peer-reviewed real-world implementations, not to estimate intervention effects or to summarize the totality of available evidence. We chose Scale for the Assessment of Narrative Review Articles (SANRA) over PRISMA (Preferred Reporting Items for Systematic Reviews and Meta-Analyses) because the work is a domain-coverage characterization rather than an evidence synthesis; structured-review reporting standards would imply dual screening, formal risk-of-bias appraisal, and exhaustive searching that we did not perform. Reporting was guided by the Scale for the Assessment of Narrative Review Articles (SANRA) [21], and abstraction of implementation features drew on DECIDE-AI (Developmental and Exploratory Clinical Investigations of Decision Support Systems Driven by Artificial Intelligence) guidance for the early-stage evaluation of decision-support systems driven by AI [22]. DECIDE-AI was developed for AI decision-support systems rather than documentation tools; we adopt its abstraction items because ambient AI scribes share the implementation features for which DECIDE-AI was designed—live clinical use, iterative product changes during deployment, embedded human-AI workflow, and the need for safety monitoring under real-world conditions. We do not import DECIDE-AI’s outcome categories wholesale, since its decision-support emphasis on diagnostic accuracy does not map directly onto documentation evaluation. Throughout, we use “prominent implementation reports” to mean publicly available, independently peer-reviewed evaluations of real-world ambient AI scribe deployments meeting all of the following: (i) deployment at a named health system or large practice (operationalized here as a multiclinic group or a named practice with a defined minimum number of clinicians, a threshold chosen to exclude micropilots and single-clinic demonstrations while retaining such practices, rather than a single-site pilot without organizational identification; where clinician count was not reported, multiclinic or enterprise-deployment language served as a proxy); (ii) reporting of at least 1 quantitative outcome relevant to documentation burden, after-hours work, clinician burnout or satisfaction, note quality, or error profile; and (iii) sufficient methodological detail to permit characterization of evaluation domain coverage. This definition is operationalized by the selection criteria for illustrative implementations below.

We searched PubMed, Google Scholar, and Scopus on several occasions, with the final search for this revision completed in April 2026. Search terms combined the concept of ambient AI scribes (“ambient AI scribe”, “digital scribe,” “AI-powered scribe,” “ambient documentation,” “generative AI documentation”) with the concepts of clinician outcomes (clinician OR physician OR “health professional”), evaluation (“implementation,” “evaluation,” “documentation time,” and “burnout”), and clinical setting (“health system,” “health care,” “primary care,” and “specialty care”). Searches were limited to English-language items published through April 2026. Results were supplemented by hand-searching reference lists of included articles, organizational websites of major health systems publicly reporting deployments, and independent evidence scans. Selection criteria for illustrative implementations required (i) a real-world implementation of an ambient AI scribe in clinical practice; (ii) reporting of at least 1 quantitative outcome relevant to documentation burden, after-hours work, clinician burnout or satisfaction, note quality, or error profile; and (iii) sufficient methodological detail to permit characterization of evaluation domain coverage. We excluded purely simulation-based studies, vendor case studies without independent peer review, and editorial commentary without primary data. Searches identified records after deduplication; these underwent full-text screening, and 6 met all selection criteria. The smaller number reflects strict requirements for independent peer review and detailed outcome reporting; a larger pool of studies cited elsewhere in this viewpoint informed the synthesis but did not meet criteria for inclusion in Table 1. A single reviewer (VSME) performed selection and extraction without dual screening or formal quality assessment. Because the goal was to illustrate evaluation patterns rather than estimate effects, we used single-reviewer screening with conservative selection criteria and a prespecified structured extraction grid; we describe operational definitions in the next paragraph and provide the full extraction grid as Supplementary Material. Missed studies remain possible and this is acknowledged as a methodological limitation.

**Table 1.** Evaluation domains assessed in major ambient AI scribe implementations^a^.

Implementation	Setting, scale, and duration	Efficiency	Satisfaction	Communication	Narrative	Reasoning	Equity
Tierney (Permanente) 2024 [23]	Integrated delivery system	Yes^b^	Yes	Subj.^c^	No^d^	No	No
Tierney (Permanente) 2025 [24]	Integrated delivery system; approx. 2.5 million encounters; approx. 12 months	Yes	Yes	Subj.	Subj.	No	No
Olson et al 2025 [7]	Multicenter quality improvement study in ambulatory settings	Yes	Yes	No	No	No	No
Liu et al. 2024 [25]	Nonrandomized clinical trial	Yes	Yes	No	Subj.	No	No
Davis et al. 2026 [26]	University of Rochester large academic medical center, outpatient care, 49 providers across 9 departments	No	No	Subj.	No	No	No
Evans et al (Australian Allied Health) 2025 [27]	Australian allied health private practice; clinician and patient perspectives	Yes	Yes	No	No	No	No

a The threshold for Yes (systematically assessed) is the use of a validated patient-reported instrument, an objective system-derived measure, or a structured audit against reference notes or transcripts. For Communication, this means the Communication Assessment Tool, the Consumer Assessment of Health Care Providers and Systems (CAHPS) Clinician and Group communication composite, or Roter Interaction Analysis System-coded analysis; for Satisfaction, the Maslach Burnout Inventory, Mini-Z, or the Stanford Professional Fulfillment Index; for Narrative, the Physician Documentation Quality Instrument (PDQI-9) or a structured audit; for Efficiency, the electronic health record audit-log time-on-documentation. Subj. denotes subjective perceptions reported through clinician self-report, single-item questions, or open-ended comments without instrumented measurement. No denotes absence of public reporting and does not prove absence of unaffected performance or of internal evaluation. General patient experience measures (overall satisfaction, perceptions of physician computer use during visits) are reported separately in the Documented Benefits text and should be distinguished from validated communication assessment; the latter is what the Communication column captures. Borderline cases were classified conservatively: unvalidated local surveys were classified Subj.; proprietary vendor scores without disclosed rubric were classified No; ambiguous instrument provenance was classified Subj. Full classification rules are detailed in Methods and in the Supplementary Material.

b Yes: systematically assessed with validated instruments or objective measures.

c Subj: subjectively assessed through unstructured self-report or single-item perceptions.

d No: not assessed in published reports.

We categorized reported outcomes in each domain according to operational definitions established before Table 1 was populated. An outcome was classified as systematically assessed when measured using a validated instrument (such as the Communication Assessment Tool, Consumer Assessment of Health care Providers and Systems (CAHPS) communication composite, Maslach Burnout Inventory, or Mini-Z), an objective electronic health record-derived measure (such as audit-log documentation time), or a structured audit against reference notes or transcripts. An outcome was classified as subjectively assessed when reported through unstructured clinician self-report, single-item perceptions, or open-ended comments without instrumented measurement. An outcome was classified as not assessed in published reports where the publication did not report any measure of that domain. Borderline cases were classified conservatively: a locally developed 3-item survey without published psychometric properties was classified as subjective rather than systematic; a vendor-dashboard “note quality score” without disclosed scoring rubric was classified as not assessed for the relevant domain, since absence of methodology prevented interpretation; where instrument provenance was unclear, the outcome was classified as subjective. Absence of public reporting does not prove absence of internal evaluation by implementing organizations. A second reviewer was not available for adjudication; this limitation is acknowledged.

Implementation features were abstracted to inform a design and reporting features table (Table 2) detailing study design, comparator, follow-up duration, vendor, setting, outcome ascertainment method, and whether patient-level data were collected. We do not claim to apply a formal risk-of-bias instrument; the table summarizes design and reporting characteristics relevant to interpretability. Abstraction items drew specifically from DECIDE-AI guidance on context of use, workflow integration, human factors, and safety monitoring under live clinical conditions, and outcome ascertainment, providing a structured basis for interpreting the evaluation domain coverage shown in Table 1 [22]. To motivate an expanded evaluation framework, we synthesized these implementation patterns with established literature on clinician burnout [28], patient-clinician communication measurement [29], narrative medicine, automation bias [15], and health equity in speech recognition [30]. This integrative approach aimed to identify systematic measurement gaps, explain why unmeasured domains may affect patient safety and care quality, and propose feasible research priorities for active deployments.

**Table 2.** Design and reporting features for the studies in Table 1.^a^

Implementation	Study design	Comparator	Follow-up	Vendor	Data source	Patient-level data
Tierney (Permanente) 2024 [23]	Pre- and postimplementation cohort	Predeployment baseline	10 weeks	Abridge (proprietary)	EHR^b^ audit logs; clinician survey	No
Tierney (Permanente) 2025 [24]	Longitudinal observational cohort	Predeployment baseline	Approximately 12 months	Abridge (proprietary)	EHR audit logs; clinician survey	No
Olson et al 2025 [7]	Multicenter quality improvement study	Preimplementation baseline	1 month	Multiple (mixed)	Clinician survey	No
Liu et al 2024 [25]	Nonrandomized clinical trial	Predeployment baseline	5 weeks	Single proprietary	EHR audit logs; clinician survey	No
Davis et al 2026 [26]	Retrospective provider-level pre- and postcohort	Each provider’s pre-DAX^c^ baseline; no concurrent control	Variable postuse period from first use in March–July 2024 through December 2024	Nuance Dragon Ambient eXperience	Epic Signal encounter counts and provider-level aggregate Press Ganey data	No
Evans et al (Australian Allied Health) 2025 [27]	Mixed methods (survey and interviews)	Within-clinician comparison	Several months	Proprietary AI scribe	Clinician survey; patient interviews	Limited

a Vendor names are reported as published; some implementations involved multiple vendors or do not name a vendor publicly. “Patient-level data” indicates whether outcomes were measured at the patient encounter level (Yes), only at the clinician level (No), or partially (Limited—eg, subset of patient interviews). All included studies are at moderate-to-high risk of bias for clinical effectiveness conclusions because of pre-post or single-arm design, absence of blinding, and short follow-up; this risk profile is associated with early-stage implementation evaluation [31] and is consistent with the generally low methodological quality identified by the recent systematic review and meta-analysis of AI documentation tools [9].

b EHR: electronic health record.

c DAX: Dragon Ambient Experience.

### Documented Benefits

Implementation studies report efficiency benefits across diverse settings, although effect sizes vary. Table 1 summarizes evaluation patterns across 6 implementation studies [7,23-27] published between 2024 and 2026. Table 2 reports the corresponding design and reporting features—study design, comparator, follow-up duration, vendor, setting, and data source—enabling readers to judge whether absent domain coverage reflects absent measurement or absent reporting.

Reported magnitudes are heterogeneous. Kaiser Permanente’s deployment across more than 7000 physicians reportedly saved nearly 16,000 documentation hours over 10 weeks, with 70 percent of users reporting reduced burnout [23]. A multisite study across 5 US academic health systems, by contrast, reportedly found modest reductions in total electronic health record time and documentation time alongside a slight increase in weekly visit volume after AI scribe adoption [8], an effect size more in line with the moderate pooled estimates of recent meta-analytic synthesis [9]. Other systems reported significant decreases in burnout measures, with substantial methodological heterogeneity in instrumentation: studies used the Maslach Burnout Inventory, the Mini-Z single-item burnout question, the Stanford Professional Fulfillment Index, and ad hoc single-item measures, and reported a mix of absolute and relative changes. Cross-study numeric comparison should accordingly be made with caution [7]. These findings replicate across organizations, specialties, and practice types, with reproducibility and modest-to-moderate effects more consistent than uniform large benefits [32]. Patient experience data indicated modest benefit. Patient satisfaction scores showed small improvements in some studies [26].

Evidence quality varies substantially by clinical setting. Peer-reviewed evaluations are concentrated in ambulatory settings, with many in primary care and outpatient internal medicine [32], where encounter structure tends to be predictable and information transfer is a substantial proportion of documentation work. Evidence from specialty care is mixed and considerably thinner, with implementations in pediatrics, oncology, and allied health practice reporting more variable benefits and identifying specialty-specific challenges including management of psychosocial complexity [33-35]. The case for evaluating communication and narrative outcomes is strongest in specialties where lifestyle counseling, chronic disease management, and emotional or psychosocial content occupy the center of the encounter rather than its margins.

Taken together, these reports describe efficiency gains that are real but heterogeneous, modest to moderate in pooled magnitude, and supported by an evidence base in which study quality remains a notable limitation. The case for continued implementation rests on this evidence; the case for systematic evaluation rests on the same evidence and on the question of whether the additional domains addressed below remain unmeasured at scale.

### Systematic Measurement Gaps

Tables 1 and 2 reveal a consistent pattern in evaluation approaches across the included publicly reported implementations. All 6 studies [7,23-27] systematically measured time savings or clinician satisfaction, establishing consistent evidence for efficiency and well-being benefits within their methodological constraints. Within this illustrative sample of 6 independently peer-reviewed, real-world implementation evaluations, none systematically assessed communication quality using validated instruments such as the Communication Assessment Tool or CAHPS communication composites. None evaluated narrative content preservation through structured comparison of AI-generated versus human documentation. None examined clinical reasoning effects through longitudinal tracking or automation bias assessment. None stratified performance by patient primary language, demographic characteristics, or encounter complexity. These observations are bounded by our selection criteria and definitions and should not be read as field-wide empirical claims; absence in this sample reflects absence of public reporting in independently peer-reviewed, real-world implementation evaluations meeting our criteria, rather than proof that no implementing organization has measured these dimensions internally.

While some studies report clinicians feeling “more present” or note modest gains in patient satisfaction, these subjective, self-reported impressions are not a substitute for rigorous assessment, given that clinician and patient ratings of communication often show poor agreement [36]. They may represent valuable preliminary signals suggesting positive communication effects. However, subjective impressions without structured assessment provide limited evidence about whether the specific communication dimensions that predict adherence, outcomes [37], and malpractice risk are preserved or affected. Two studies reported subjective perceptions of documentation quality or noted high retention rates of AI-generated content in final notes [20,38], but these assessments differ from a structured comparison examining whether narrative elements essential for care coordination and clinical reasoning are consistently preserved.

Error analyses were provided concerning preliminary data. Biro et al [10] conducted an instrument-validation study comparing AI-generated drafts against reference standards under controlled conditions; this is not a real-world implementation study and findings are not directly generalizable to active clinical practice. Within those constraints, the audit found that AI-generated drafts contained errors that must be evaluated to mitigate safety risks. The meta-analytic synthesis reported that note quality was at least comparable to that of manually prepared notes [9], indicating that aggregated comparison may attenuate the per-note error rates seen in instrument-validation work. Evidence from multilingual encounters, family-mediated consultations, and interpreter-assisted visits was notably absent across both implementation studies and the broader meta-analytic literature.

### Why Additional Domains Warrant Investigation

#### Communication Quality as Foundation of Clinical Effectiveness

Patient-clinician communication fundamentally shapes health care quality. Communication affects diagnostic accuracy through information disclosure completeness [39], treatment adherence through shared decision-making quality [40], and patient satisfaction through perceived caring and partnership [37]. Meta-analyses demonstrate that better communication correlates with improved adherence, improved health outcomes, and higher satisfaction [29,41]. The Joint Commission identifies communication failures among the leading causes of sentinel events [42].

No implementation study in our included sample measured communication quality using validated instruments capable of detecting effects in either direction, leaving this question unresolved within the evidence base most directly relevant to active deployments. In non-AI contexts, evidence regarding recording effects is mixed. Studies examining video recording for research purposes found both minimal impact and meaningful changes in interaction patterns, with effects varying by context [43].

The presence of recording might degrade communication if patients become guarded in disclosure or if clinicians reduce empathic responses knowing these typically do not require documentation. Conversely, recording might improve communication if clinicians, freed from the mental load of concurrent documentation, provide more attentive listening and empathic responses. Patient experience data showing increased perception of physician attention suggests this positive pathway may predominate in many encounters [44]. However, preliminary positive signals from satisfaction surveys, though valuable, differ from systematic assessment using instruments designed to detect subtle changes in communication dimensions that predict clinical outcomes.

The tools exist and are feasible to deploy. Validated measurement of patient-clinician communication operates at 3 levels: multi-item patient-reported scales (the Communication Assessment Tool, a 14-item patient-rated measure of physician communication developed and tested by Makoul et al [45]; the CAHPS Clinician and Group communication composite, the canonical patient-reported measure for interorganization benchmarking, maintained by the Agency for Health Care Research and Quality [46,47]); observational coding such as the Roter Interaction Analysis System [48]; and encounter-level behavioral proxies derived from audit logs or recordings. The Communication Assessment Tool produces clinician-specific feedback at the visit level and is well suited to local quality improvement [49]; the CAHPS CG communication composite serves as the standard for system-level benchmarking and is distinct from CAHPS general-satisfaction items [50]; observational coding captures interactional patterns invisible to either patient-reported instrument. Local short-form instruments and conversation-analytic methods have a place but produce data less suited to interorganization comparison. The point is not that only the Communication Assessment Tool or CAHPS counts; it is that subjective single-item perception is not equivalent to validated communication measurement, and that pragmatic, deployable instruments already exist. These instruments could be incorporated into ongoing implementations to determine whether preliminary positive signals reflect actual communication quality preservation or improvement, or whether they coexist with subtle degradation in dimensions that general satisfaction questions do not capture.

#### Narrative Preservation and Care Coordination

Clinical documentation preserves more than discrete biomedical facts. It captures temporal symptom patterns, patient explanatory models, psychosocial context, and narrative coherence, enabling subsequent providers to understand what documented information means for patient care [51]. Research on electronic health record effects found that structured templates, while improving data capture, often degraded narrative coherence, with clinicians reporting that template-driven documentation obstructed building the patient’s story [52].

Error analyses indicating frequent omissions in AI-generated drafts under instrument-validation conditions [53] raise concerns about what narrative elements may be systematically lost at scale. Measurement of narrative preservation can proceed in stages. The Physician Documentation Quality Instrument (PDQI-9) provides a starting point: it assesses note quality across 9 dimensions including up-to-date accuracy, thoroughness, usefulness, organization, comprehensibility, succinctness, synthesis, and internal consistency [54] and has been used in prior studies of electronic health record note quality [55]. PDQI-9 fits best as a baseline tool for outpatient consultation notes and is less well suited to high-tempo settings: a study in emergency medicine found that PDQI-9 did not adequately discriminate scribe-note quality, suggesting that adaptation or complementary instruments may be needed for emergency documentation, structured discharge summaries, and consult-to-transfer notes [56]. PDQI-9 does not, in addition, capture the narrative-specific features most at risk under automated documentation, temporal coherence, patient explanatory model, psychosocial context, and care-coordination utility, each of which would benefit from a validated extension or a supplementary scale developed for this purpose. A staged research program would therefore: first, deploy PDQI-9 in comparative studies of AI-generated and pre-AI clinician documentation to establish baseline note-quality differences using existing validated tools; second, develop and validate narrative-specific extensions for the 4 features above; and third, conduct large-scale comparative evaluations using the combined instrument set.

Methodological development must address the comparator problem directly. The relevant question is not whether AI-generated documentation matches an idealized researcher transcript, but whether it preserves narrative content as well as, or better than, the manual documentation it replaces. Manual documentation has its own well-documented gaps, including omissions and inconsistent documentation [57], and equity-relevant disparities in how patients of different backgrounds are described [58]. Validated rubrics should accordingly compare AI output against multiple references, pre-AI manual documentation by the same clinician, peer-clinician documentation, and verbatim transcripts, to distinguish AI-specific failure modes from baseline documentation limitations.

#### Cognitive Engagement and Skill Development

Documentation differs from clinical decision support in important respects. Decision-support automation bias concerns clinicians accepting an AI recommendation about a discrete clinical decision; documentation automation bias concerns clinicians accepting an AI synthesis of an encounter that itself shapes downstream clinical reasoning. The translation between domains is not trivial: documentation produces a narrative artifact rather than a discrete recommendation; the time-pressure under which review occurs differs; and the failure modes (omission and silent restructuring) are qualitatively unlike an incorrect alert. Even so, the underlying cognitive mechanisms—reliance, overtrust, and reduced critical engagement with an authoritative-looking automated output—plausibly generalize. Evidence from the decision-support context, while not directly transferable, illuminates the mechanism. An empirical study of prescribing decision support reports that clinicians overrode correct initial judgments in approximately 5% of prescribing cases when presented with erroneous automated advice [59]. Recent evidence from large language model diagnostic assistance found physicians using ChatGPT (OpenAI) performed no better than those without AI access despite ChatGPT alone outperforming physicians on the same cases, suggesting superficial engagement with AI outputs [60].

Skill degradation with prolonged automation reliance appears in aviation, surgery, and other domains [61,62]. A multicenter observational study of physicians using AI assistance showed performance degradation when AI was withdrawn, suggesting dependence development rather than skill augmentation [63]. Documentation represents more than transcription—it is where clinical reasoning crystallizes from observations into coherent assessments. If clinicians accept AI documentation outputs without comparable engagement, 3 specific outcomes would demonstrate cognitive offloading rather than helpful scaffolding: trainee-generated notes on standardized cases of lower quality than those of historical comparator cohorts; declining diagnostic-reasoning performance when AI assistance is withdrawn (the AI-off probe used in adjacent literature [64]); and inverse correlation between AI exposure intensity and standardized reasoning assessments at program exit. Conversely, if AI assistance supports skill development, the same instruments would show preserved or improved performance.

The balance between helpful scaffolding and harmful cognitive offloading likely depends on implementation approach, supervision structures, and whether learners are taught critical engagement with AI outputs. No longitudinal studies compare skill trajectories of trainees with varying AI exposure [65,66], warranting prospective assessment in institutions recently adopting AI scribes.

#### Performance Equity Across Populations

Equity in clinical documentation is not a problem ambient AI scribes introduce. Studies of manual documentation have revealed disparities, with patients from minority backgrounds more likely to be described in stigmatizing terms [58]. The relevant question for AI scribes is therefore whether they amplify, attenuate, or shift these existing patterns, and whether efficiency gains generalize across populations or accrue disproportionately to some.

Most published AI scribe evaluations have examined primarily English-speaking patients in straightforward encounters [67,68]. Performance in multilingual consultations, interpreter-mediated encounters, and family-involved decision-making remains only partially examined despite these being common in diverse health care settings globally. In Asian health care contexts, including Singapore, multilingual encounters involving Mandarin, Cantonese, Tamil, Malay, and English code-switching represent normative practice rather than exceptional circumstances [69], with family members routinely participating in medical decision-making across generations [70]. Clinical experience in such settings reveals specific challenges when AI systems must accurately attribute speaker identity among multiple family members, distinguish patient speech from interpreter or family translation, handle mid-sentence language switching, and preserve interactional patterns containing diagnostic information about family dynamics and care preferences.

Algorithmic bias and disparate performance across demographic groups are well documented in other health AI applications [71]. Speech recognition systems demonstrate lower accuracy for speakers with nonstandard accents, minority racial and ethnic groups, and non-English languages [72,73]; in the flagship analysis, Koenecke et al [30] reported substantial racial disparities in word error rates across the 5 major commercial automatic speech recognition (ASR) systems. This is component-level evidence about the front end of the ambient-scribe pipeline rather than direct evidence of inequity in the final note. The downstream artifact reflects compounded effects from ASR, diarization, and summarization; the staged audit proposed earlier—word error rates first, speaker-attribution accuracy second, clinical concept and narrative-relevant omission third—is the analytic foundation for equity claims about full ambient-scribe systems. If AI scribes exhibit similar biases, documented benefits may not generalize to all populations and may come at the cost of disparate impacts for specific groups. When AI systems struggle with speaker attribution in family consultations involving multiple languages, attribution errors create documentation inaccuracy or require extensive clinician revision time, effectively reducing benefits for clinicians serving diverse populations.

Few published studies have stratified ambient AI scribe error rates by patient primary language, accent characteristics [74], interpreter presence, or demographic characteristics. Equity assessment can begin while methods for narrative preservation are still being developed; performance heterogeneity by language, accent, interpreter presence, and family-mediated speech can be measured with extant transcription accuracy and information extraction methods, and need not wait on the more demanding work of narrative-fidelity rubrics. Sequencing equity work after narrative methodology risks entrenching disparities during the period of fastest deployment.

#### Proposed Evaluation Framework and Research Priorities

An expanded evaluation framework assessing 4 additional domains would complement rather than replace current efficiency and satisfaction metrics: communication quality measurement using validated instruments; narrative content preservation requiring methodological development as a longer-term priority; cognitive and skill development effects, particularly in training contexts; and equity across languages and demographics. Three research priorities offer immediate feasibility for organizations with evaluation capacity, while narrative assessment requires foundational methodological work before implementation-ready tools exist. The 4 domains are parallel rather than sequential: communication, cognitive and skill development, and equity work can proceed using extant instruments, and findings in any one domain inform the others.

### Priority 1: Communication Quality Assessment

Communication quality assessment offers the highest immediate feasibility, given existing validated instruments and that many organizations already collect relevant data. Organizations implementing AI scribes could begin assessment using the Communication Assessment Tool or the Consumer Assessment of Health care Providers and Systems Clinician and Group (CAHPS CG) communication composite, both of which have established reliability and validity [75,76] for measuring dimensions of patient-clinician interaction that predict clinical outcomes [41]. The 2 instruments serve complementary purposes and can be deployed together. The Communication Assessment Tool is administered at the visit level and produces clinician-specific feedback well suited to local quality improvement [49]. The CAHPS CG communication composite is standardized for interorganization benchmarking and is a widely used patient-reported measure for system-level comparison [46]. Organizations seeking both internal improvement and external benchmarking can use both with limited additional patient burden.

Study designs should include cluster randomized trials comparing departments randomized to implement AI scribes versus traditional documentation, with communication measured at baseline and follow-up intervals. Alternatively, interrupted time series analyses can track communication scores before and after implementation using statistical controls for temporal trends and patient characteristics. Organizations already collecting patient experience data can add validated communication items, although achieving meaningful results requires biostatistical expertise that academic centers possess more readily than community settings [77]. Collaborative approaches in which academic centers lead multisite studies including community hospitals would distribute measurement burden while ensuring findings generalize across practice contexts. We propose 2 tiers for organizations with evaluation capacity. Baseline feasible: a single patient-reported communication item (eg, one CAHPS CG composite question [78]) added to existing patient experience surveys, with pre- and postimplementation comparison at clinic level, no clinician-level benchmarking required and achievable wherever an existing CAHPS or equivalent infrastructure operates. Ideal: a patient-reported Communication Assessment Tool or full CAHPS CG communication composite at the visit-level [78] with clinician-level reporting; an interrupted-time-series design at clinic-level, or—where multiple clinics roll out sequentially—a stepped-wedge design; baseline and follow-up assessments aligned with the rollout calendar; analysis adjusted for temporal trends, clinician, and patient case mix. Clinician-level sample-size targets (for example, 50 patients per clinician per evaluation period) are appropriate for benchmarking studies [79] but are not a prerequisite for system-level effect detection.

### Priority 2: Longitudinal Trainee Cohort Studies

These designs are hypothesis-testing investigations motivated by plausible mechanisms identified in the adjacent literature on documentation, decision support, and skill acquisition, rather than being responses to known safety concerns regarding accuracy and omissions in ambient-scribe practice [80]. Longitudinal skill development assessment merits high priority despite requiring extended commitment and substantial resources. Enrollment should begin immediately in training programs recently adopting AI scribes, recognizing that cohorts must progress through multiyear training before graduation outcomes can be assessed. Implementation should compare programs recently adopting AI scribes with matched programs delaying implementation, with baseline assessment at program entry establishing incoming capabilities prior to AI exposure.

Outcome measurement should include independently generated documentation quality on standardized cases (using PDQI-9 and narrative extensions where available), diagnostic reasoning capacity using validated instruments, and clinical knowledge assessment, with periodic AI-off probes to test whether reasoning performance is preserved when AI assistance is withdrawn. This approach would determine whether trainees exposed to routine AI assistance develop documentation and reasoning competencies differently from those trained without such assistance, and whether experienced clinicians maintain independent documentation skills when using AI regularly. Collaborative approaches in which multiple organizations jointly fund research would distribute costs while ensuring findings generalize across practice contexts and training environments. We propose 2 tiers for organizations with evaluation capacity. Baseline feasible: a single-program cohort enrolling trainees at program entry, with PDQI-9-scored documentation on 1 or 2 standardized cases at program entry, mid-training, and exit, AI-on assessment only; this is achievable within most academic programs without interinstitutional coordination. Ideal: prospective cohort enrolling trainees at program entry now, AI-exposed cohort versus matched programs delaying implementation; outcomes include PDQI-9-scored documentation on standardized cases and validated diagnostic-reasoning probes administered AI-on and AI-off at program entry, mid-training, and exit; follow-up to graduation, with longer-term extension where feasible. We acknowledge that a five- to seven-year follow-up horizon is unrealistic for many programs [81]; partial follow-up to early postgraduate practice is informative even where graduation-plus follow-up is not feasible.

### Priority 3: Equity and Performance Heterogeneity Analysis

Equity analysis merits immediate priority given both feasibility and ethical urgency. Implementation should focus on health care organizations serving diverse patient populations with substantial proportions of non-English encounters, interpreter-assisted visits, and patients from communities experiencing health care disparities. Primary stratifiers are: (1) patient primary language; (2) interpreter status (none, professional, family, or ad hoc); (3) accent or dialect group where feasibly identifiable; and (4) demographic categories defined by self-reported race, ethnicity, age, and gender. The minimum reporting standard for equity-aware evaluations is stratified ASR-level and content-level error rates, stratified efficiency benefits (time savings and revision burden), and stratified patient-reported experience, each accompanied by per-stratum sample sizes and confidence intervals. The study design uses deliberate oversampling ensuring adequate representation of diverse populations rather than sampling proportionate to frequency in organizational caseloads.

Blinded expert raters review AI-generated documentation, identifying errors including omissions, misattributions, and narrative gaps. Comparators should include both verbatim transcripts and pre-AI manual documentation by the same clinician and peer clinicians, so that AI-specific failure modes can be distinguished from baseline documentation limitations. Analysis is stratified by patient language, interpreter presence, and demographic characteristics, examining whether error rates differ across strata after adjusting for clinical complexity. Additionally, the assessment examines whether efficiency benefits are equitably distributed by analyzing time savings and revision burden across clinicians serving patient panels with varying diversity characteristics. Organizations serving multicultural communities could conduct such analyses with results emerging in reasonable timeframes, providing essential evidence about whether documented benefits generalize across populations or mask disparate impacts.

Community engagement with patient advisory councils and representatives from affected communities should begin before study implementation and continue throughout evaluation, ensuring that equity assessment addresses community priorities rather than only researcher-defined outcomes. Minimum specification for organizations with evaluation capacity and a diverse local catchment: deliberate oversampling targeting at least 30% non-English encounters where the catchment supports this; stratified ASR-level and content-level error rates; comparators including both verbatim transcripts and pre-AI manual documentation by the same and peer clinicians; blinded expert audit; results reported with per-stratum sample sizes and CI; community engagement throughout. Organizations without a sufficiently diverse caseload to support stratified analysis can contribute through pooled sentinel surveillance described in practical guidance.

### Practical Guidance for Implementation

#### Implementation with Embedded Evaluation

Organizations currently implementing or considering ambient AI scribes should adopt them where benefits are demonstrated locally and where implementation can be paired with prospective evaluation from initial deployment. This means establishing baseline measurement across relevant outcome domains before deployment begins, enabling before-and-after comparison or control-period data for analysis. Organizations should select evaluation priorities based on context: safety-net hospitals and practices serving diverse populations should prioritize equity validation; academic medical centers with training programs should establish trainee cohort studies; and all organizations with evaluation capacity should, at a minimum, assess communication quality given its feasibility and centrality to care quality [82].

Large academic medical centers and integrated delivery systems possess resources for comprehensive evaluation across multiple domains and should view evaluation capacity as an essential component of responsible implementation. Allocating a portion of implementation budgets for systematic evaluation ensures deployment decisions are evidence-based. Community hospitals and mid-sized practices with limited research infrastructure should focus on feasible priorities, particularly communication quality assessment using existing patient experience survey infrastructure, and participation in collaborative multisite studies that distribute research burden while contributing to field-wide evidence.

Small ambulatory practices and solo practitioners typically lack capacity for formal evaluation and should not be expected to generate independent statistical findings on quality dimensions [83]. Their contribution lies in routine ongoing monitoring (described below) and in participation in pooled or sentinel surveillance led by specialty societies and learned colleges. Organizations should commit to disseminating findings, including negative results and implementation challenges, through peer-reviewed journals, presentations at national meetings, and collaborative networks. This transparency ensures that field-wide evidence generation reflects both successful implementations and problematic patterns, enabling other organizations to make more informed deployment decisions.

### Ongoing Monitoring in Practice

Formal evaluation studies operate on multiyear timelines [84]. Clinicians and organizations using ambient AI scribes today need lighter-weight tactics for monitoring quality between research findings, and several can be adopted with minimal infrastructure. These tactics complement rather than substitute for formal research; they give clinicians a way to maintain situational awareness of their AI tools in the years before validated outcome measures and longitudinal cohorts mature.

The first tactic is periodic side-by-side comparison. Clinicians can sample a small proportion of their own encounters—a feasible target is 1 in 20 notes—and dictate or write a brief independent version of the note before reviewing the AI output. The comparison surfaces systematic omissions and stylistic drifts that pass unnoticed in routine review, particularly the subtle erosion of psychosocial context and patient explanatory models [85].

The second is targeted spot-checking. Encounters with high information density or psychosocial weight—initial consultations, disclosures of significant diagnoses, complex shared decision-making, conversations involving family members—warrant routine review of AI output against the clinician’s own recall of the conversation. These are the encounters in which omissions matter most and in which AI performance is least studied [67].

The third is structured patient feedback. Brief items added to existing patient experience surveys can capture comfort with recording technology and perceived effects on the encounter, providing a real-time signal to clinicians and managers about whether implementation is progressing effectively.

The fourth is participation in pooled surveillance. Small ambulatory practices and solo practitioners cannot generate statistically meaningful internal evaluations, but specialty societies and learned colleges can convene sentinel surveillance networks. Member practices contribute anonymized samples of AI-generated notes for centralized review by trained raters, generating system-level evidence that no single practice could produce.

### Regulatory Considerations

Current regulatory frameworks treating ambient AI scribes primarily as administrative tools [86] may inadequately address their influence on clinical documentation quality, diagnostic reasoning, and care coordination. Documentation is clinically consequential rather than purely administrative: it preserves diagnostic reasoning, enables care coordination, and provides the medical record on which subsequent decisions depend [87]. Technologies transforming documentation processes warrant regulatory attention proportionate to their potential impact on care quality and patient safety. Two reporting expectations are tractable across jurisdictions and would generate the evidence currently absent from voluntary disclosure. The first is stratified predeployment performance reporting: word error rate, speaker attribution accuracy, and clinical concept omission rates reported separately by patient primary language, demographic group, encounter complexity, and, where feasible, accent or dialect group, rather than aggregate accuracy alone. The second is postmarket monitoring with disclosure: ongoing reporting of revision burden, vendor-side updates to underlying models, and signal-level performance changes following retraining or model swap. Both expectations sit at the vendor level, where data are routinely collected; equivalent requirements on individual clinicians’ documentation practices would not be feasible and would not generate the systematic evidence the field requires.

Regulatory taxonomies differ materially across jurisdictions in how the administrative-tool versus clinical-decision-support boundary is drawn [88] and in the instruments through which postmarket surveillance is required [89]. The principles above are portable; the regulatory instruments through which they are applied—United States Food and Drug Administration software-as-a-medical-device pathways, the European Union AI Act, the United Kingdom Medicines and Health Care Products Regulatory Agency framework, Singapore’s Health Sciences Authority guidance, and other regional regimes [90],—are not. Regulation must balance evaluation requirements against genuine workforce sustainability benefits, avoiding overly restrictive approaches that would harm clinician well-being and health care delivery capacity.

## Discussion

Ambient AI scribes deliver generally promising but heterogeneous efficiency and clinician well-being benefits [91]. Pooled effect sizes from recent meta-analytic synthesis are moderate; multisite evaluation describes modest reductions in electronic health record and documentation time, and study quality across the underlying evidence base is generally low [9]. These benefits are real, replicated across diverse settings, and clinically meaningful for workforce sustainability; they are also smaller and more variable than initial single-system reports suggested. Current evaluations focus narrowly on time savings and satisfaction [92], leaving critical domains unmeasured. This analysis identified systematic evaluation gaps concerning patient-clinician communication, narrative content preservation, cognitive effects on clinical reasoning, and equity. We frame these as plausible, measurable hypotheses warranting investigation, not as harms established for ambient scribes; the case for evaluation rests on adjacent evidence and on the scale of deployment, not on demonstrated harms specific to these systems.

This evaluation pattern may be appropriate for early-stage technology assessment, with efficiency and satisfaction metrics correctly prioritizing the establishment of feasibility and user acceptance as a foundation for broader deployment. However, as adoption scales from pilot projects to enterprise-wide deployment and integration into training programs, evaluation frameworks must mature to encompass comprehensive care quality assessment. The question is not whether documented benefits justify initial adoption but whether continued rapid expansion should proceed with or without evidence regarding additional care quality dimensions.

The conceptual framework (Figure 1) clarifies how ambient AI scribes may produce both positive and negative effects through 2 primary pathways: recording-device presence during encounters and automated draft generation. Each pathway has plausible benefits and risks for communication, narrative, reasoning, and equity. Recognition of competing pathways emphasizes that neither universally positive nor universally negative conclusions are likely accurate and that effects probably vary by context, implementation approach, and population.

For clinicians and health systems, demonstrated well-being benefits support continued adoption in settings where they are observable locally [7], paired with quality safeguards including structured audits of AI-generated notes, measuring patient-clinician communication with validated instruments where feasible, and developing protocols for use in complex or multilingual encounters. For medical education, the critical challenge is balancing efficiency gains for trainees with developing independent documentation and reasoning competencies. Documentation differs from clinical decision support: the AI-off probe used in adjacent literature provides a concrete way to detect skill degradation in trainees and would be a productive endpoint for prospective trainee studies [93]. Policymakers and developers must prioritize equitable performance across patient populations, evaluating ambient AI scribes against both transcript reference standards and preAI clinician documentation, because speech-recognition disparities may interact with preexisting inequities in clinical documentation and patient description [19,94].

This work has several strengths, including synthesizing findings from prominent, large-scale, recent ambient AI scribe implementations and integrating recent multisite and meta-analytic evidence on the magnitude and quality of efficiency benefits. By drawing upon established literature from communication science, medical education, automation psychology, and health equity, it provides robust conceptual grounding for proposed evaluation frameworks. The research agenda is pragmatic, leveraging existing validated instruments and feasible study designs integrable into current implementations.

However, limitations warrant acknowledgment. As an analytic viewpoint rather than a systematic review, this analysis is subject to selection bias and may not capture the entirety of rapidly evolving literature; reporting was guided by SANRA principles to maximize transparency within these constraints, but selection and extraction were performed by a single reviewer (VSME). Conclusions rest on studies with often short follow-up periods and generally low methodological quality, limiting insight into potential long-term effects. Critically, concerns raised about automation bias, deskilling, and narrative degradation remain plausible risks grounded in adjacent literature rather than demonstrated harms of ambient AI scribes specifically. This analysis proposes hypotheses requiring testing rather than documenting established harms that necessitate immediate intervention. Empirical investigation may reveal that theoretical concerns do not manifest clinically or that positive pathways predominate.

The path forward involves launching rigorous communication quality studies, establishing longitudinal cohorts assessing cognitive and educational impacts, and conducting dedicated audits ensuring equitable performance. By embracing broader evaluation frameworks, the field can ensure ambient AI scribes not only restore time to clinicians but also robustly support the quality and humanity of patient care. The opportunity to generate this evidence is time-limited, as adoption expands and practice patterns develop [95].

## Conclusions

Ambient AI scribes show efficiency and some clinician well-being benefits, but evidence remains limited, heterogeneous, and context-dependent [7,91]. Current evidence supports continued implementation with quality safeguards in settings where benefits are demonstrated locally, rather than uniform adoption justified by early single-system reports. Existing evaluation frameworks focus narrowly on time savings and satisfaction while leaving questions about quality of care, equity, and other downstream impacts systematically unmeasured [6].

An expanded framework would complement existing approaches with 3 near-term priorities: validated communication measurement deployable within ongoing rollouts; longitudinal trainee cohort studies launched at programs recently adopting AI scribes, including AI-off probes to test for skill degradation; and stratified equity analyses oversampling multilingual and interpreter-mediated encounters, with comparators including both verbatim transcripts and preAI manual documentation. Narrative preservation can advance in stages, beginning with PDQI-9 and progressing to validated narrative-specific extensions [54,96]. In parallel, organizations and clinicians can adopt lighter-weight monitoring tactics—periodic side-by-side comparison, targeted spot-checking, structured patient feedback, and pooled sentinel surveillance—to maintain situational awareness during the years before formal evidence matures.

The goal is not to slow adoption but to ensure that it proceeds with evidence commensurate with scale. The window for generating that evidence is narrowing as adoption expands and practice patterns develop [97]. Three audiences have specific next steps. Editors and reviewers can require consistent domain coverage reporting for ambient AI scribe evaluations submitted for publication. Implementing organizations can preregister evaluation plans across the 4 domains before rollout, select priorities according to local context and patient populations served, and commit to publishing findings including negative results. Vendors can publish stratified performance data and postmarket monitoring signals as a routine condition of deployment.

## Abbreviations

ASR: automatic speech recognition
CAHPS: Consumer Assessment of Health Care Providers and Systems
CAHPS CG: Consumer Assessment of Health Care Providers and Systems Clinician and Group
DECIDE-AI: Developmental and Exploratory Clinical Investigations of Decision Support Systems Driven by Artificial Intelligence
PDQI-9: Physician Documentation Quality Instrument
PRISMA: Preferred Reporting Items for Systematic Reviews and Meta-Analyses
SANRA: Scale for the Assessment of Narrative Review Articles
